# Latitudinal variation in seagrass communities with special emphasis on post-tsunami status in the Andaman and Nicobar archipelago, India

**DOI:** 10.1371/journal.pone.0300654

**Published:** 2024-03-20

**Authors:** Swapnali Gole, Nehru Prabakaran, Sumit Prajapati, Sohini Dudhat, Himansu Das, Sivakumar Kuppusamy, Jeyaraj Antony Johnson

**Affiliations:** 1 Wildlife Institute of India, Dehradun, Uttarakhand, India; 2 Marine Threatened Species and Habitats, Terrestrial & Marine Biodiversity, Environment Agency, Abu Dhabi, UAE; King Abdulaziz University, SAUDI ARABIA

## Abstract

We studied spatial variation in seagrass communities in the Andaman and Nicobar archipelago (ANI), India using latitude as a surrogate variable. We classified the ANI into five latitudinally distinct island groups: North & Middle Andaman, Ritchie’s archipelago, South Andaman, Little Andaman, and the Nicobar archipelago. We evaluated the Importance Value Index (IVI) for species to determine the ecologically dominant seagrasses within each Island group. Later, we related our findings to investigate the three decadal pre- and post-tsunami status of seagrass habitats in the ANI which were severely impacted by the Indian Ocean tsunami of 2004. Six of the 11 observed species, such as *Halophila ovalis*, *Halophila beccarii*, *Halophila minor*, *Halodule pinifolia*, *Thalassia hemprichii*, and *Cymodocea rotundata*, dominated the seagrass population among all island groups. Seagrass composition significantly varied across the five investigated latitudinal gradients. Seagrass communities in ’Ritchie’s Archipelago and Nicobar’ and ’South Andaman and Little Andaman’ revealed the highest and lowest variation. Further, Ritchie’s Archipelago and Nicobar had the highest species richness (n = 10), followed by North & Middle Andaman (n = 8), and the lowest in South and Little Andaman (n = 6). Despite similar species richness and composition, Nicobar contributed to the highest seagrass coverage compared to the lowest recorded in the Ritchie’s Archipelago. Our observations on the re-colonization of disturbed areas by early successional and historical species suggest recovery of the seagrass population in the ANI post-disturbance. Lastly, co-variates associated with latitude as a surrogate warrant further investigation.

## Introduction

Species diversity and distribution are a product of complex interactive processes fundamentally driven by several biotic and abiotic factors [1]. These interactions vary across different spatial scales [2]. The latitudinal scale and its influence on species diversity have intrigued the scientific communities for a long time [2–4]. Species richness is known to decline from the tropics to the poles [5]. Spatial heterogeneity [6], tropic-level interactions, energy availability [7], high rates of speciation [8], and geological stability [9] in tropical habitats over polar regions mainly influence the large-scale latitudinal variation in species richness. In the aquatic systems, the latitudinal trends and trait dispersion in community ecology furthermore, are significantly observed in marine waters than in freshwater systems [2,10].

Like other ecological groups, seagrasses exhibit a latitudinal variation in species diversity [11]. The tropical Indo-Pacific Bioregion harbors the maximum number of seagrass species (n = 24) [12], with > 50% of these species co-occurring in mixed seagrass meadows [11]. Further, this region supports four (out of five) high seagrass diversity regions of the World [12]. A decreasing trend is observed in species richness from this tropical seagrass hotspot towards higher latitudes [11]. In addition, seagrasses being a polyphyletic group [13], the Indo-Pacific bioregion is a hypothetical center of seagrass species origin [11]. Thus, the tropical Indo-Pacific bioregion forms one of the critical seagrass biomes with evolutionary significance.

In the current scenario, like worldwide threats to seagrasses, the Indo-Pacific bioregion shows an alarming decline in seagrass meadows [14]. In conjunction with human-induced causes, natural disasters like hurricanes, cyclones, and tsunamis have severely impacted seagrasses, altering habitats and species composition [15–17]. The December 2004 Sumatra-Andaman Earthquake (Mw 9.3) was one such critical catastrophe in the recent past, that triggered a massive trans-oceanic tsunami in the North Indian Ocean, resulting in coastal destruction in the mainland and continental India [18]. The collective impact of the tsunami was significantly observed on seagrasses in the Andaman Sea [19,20], including the Andaman & Nicobar archipelago (hereafter ANI) of India [21]. The MSK intensity of the earthquake faced by ANI was so intense, that it led to land drift of the Islands, coastal uplift in the North Andamans (MSK intensity VII) and coastal subsidence in the South Andaman and the Nicobar Islands [MSK intensity VIII; 18,22]. Alarming observations on the ecosystem damage revealed enhanced turbidity levels, nutrient loading, and loss of primary productivity. The magnitude of destruction, as stated by Thakkar & Goyal [23], "Strong coastal erosion has created sea caves," sheds light on the scale of damage incurred by high-value coastal habitats in ANI, including seagrasses [21].

Habitat destruction sustained by the coastal seagrass communities due to catastrophes like the tsunami may have had cascading effects on the local populations in the impacted regions. Thus, studying this change and the spatiotemporal trends that will contribute to seagrass biodiversity management and conservation is imperative. Seagrass species distribution and recovery depend on species-environment interactions [24] and species’ growth strategies to recolonize [25]. For a region like the ANI that latitudinally spreads across a coastline of 1962 km (www.andaman.gov.in), a local-scale variation in predictor variables further influences seagrass distribution. In addition, the magnitude of natural stressors, such as cyclonic storms and tsunamis, impacting the ANI coastline differ significantly [26]. In the latter catastrophe, the Nicobar archipelago faced more massive devastation than the Andaman archipelago due to the former’s geographical closeness to the earthquake’s epicenter (180 kms from Great Nicobar) [23,27]. Subsequent impacts were observed in gradients for coral reefs [28] and mangroves [29], where the distance from the disaster was proportionate to the effects suffered.

Here, latitude can act as a surrogate variable to understand the spatiotemporal trends in seagrass communities along a diverse coastline of ANI. Thus, this study aimed to assess the present status of seagrasses in the ANI across latitudinally distinct island groups. Further, we delved into comparing the current findings with the pre-tsunami (before 2004) and recent seagrass baseline surveys to investigate the seagrass recovery status in the ANI. The ’scale of analysis’ is an essential attribute for a comparable latitudinal variation in species richness, referring to the study’s sampling unit and spatial extent [4]. Uniformity in these attributes between two or more sampled sites allows ecological comparisons across spatial scales. In the present study, we used a similar approach, keeping the sampling unit consistent and the spatial extent of surveys comparable across the surveyed latitudinal gradient.

## Materials and methods

### Ethics statement

We obtained all required permits for fieldwork from the Department of Environment and Forests, Van Sadan, Chatham (Port Blair). In addition, we procured boat permits from the Directorate of Fisheries to carry out SCUBA-aided surveys using a fishing vessel. To enter the tribal protected areas of Central Nicobar, we attained authorization (tribal pass) from the Deputy Commissioner, South Andaman. Additional to the tribal pass, we took verbal consent from the Head of the tribal council of each village in Nicobar before entering tribal protected waters. Prior to all field-based surveys, the Divisional Forest officers, Station head officers of police departments, and defence patrolling agencies (Indian Navy and Indian Coastguard) based in each island, were intimated about the fieldwork.

### Study site

The Andaman and Nicobar archipelago (ANI), India, is in the Bay of Bengal, off the eastern coast of mainland India. Spread across a vast latitudinal gradient (6° 45’ 39.13" N to 13° 39’ 43.77" N), the coastline of ANI is highly indented [30]. Across the spatial scale, the ANI experiences inconsistent local climate regimes, where rainfall in the island exhibits a latitude driven trend. Average annual rainfall varies from 290 cm to 264 cm for the Andaman archipelago, and the Nicobar archipelago, respectively [31]. However, for all seasons except monsoon (June to September), Little Andaman and Nicobar Islands experience higher rainfall than the Andaman group, due to higher strength of easterlies at lower latitudes [31]. Contrary, in monsoon, lower-level westerlies have a stronger influence on the Andaman group than Little Andaman and Nicobar, resulting in discrepant higher rains [60% in Andaman as opposed to 40% in Nicobar; 31].

Thus, latitudinal differences in the Islands exert micro-climatic variations, which are critical in influencing the local environment and biodiversity. Resultant, the biodiversity also varies latitudinally in ANI, contributing to two distinct global biodiversity hotspots; Indo-Burma and Sundaland. The Northern part of the ANI (Andaman) is placed under the Indo-Burma Biodiversity hotspot, and the Southern group of Islands (Nicobar) forms a part of the Sundaland Biodiversity hotspot [32]. Mangroves, salt marshes, coral reefs, and seagrasses form some of the crucial marine ecosystems of the coastal waters in the ANI [33]. Broadly, ANI is divided further as the Andaman archipelago and the Nicobar archipelago, separated by the ten-degree (latitude) channel. We established our field surveys across five geographically distinct Island clusters, each representing a different latitudinal gradient such as; 1) The North and Middle Andaman (N&MA), 2) The Ritchie’s archipelago (RA), 3) The South Andaman (SA), 4) The Little Andaman (LA) and 5) The Nicobar Islands (NIC).

**Fig 1:**
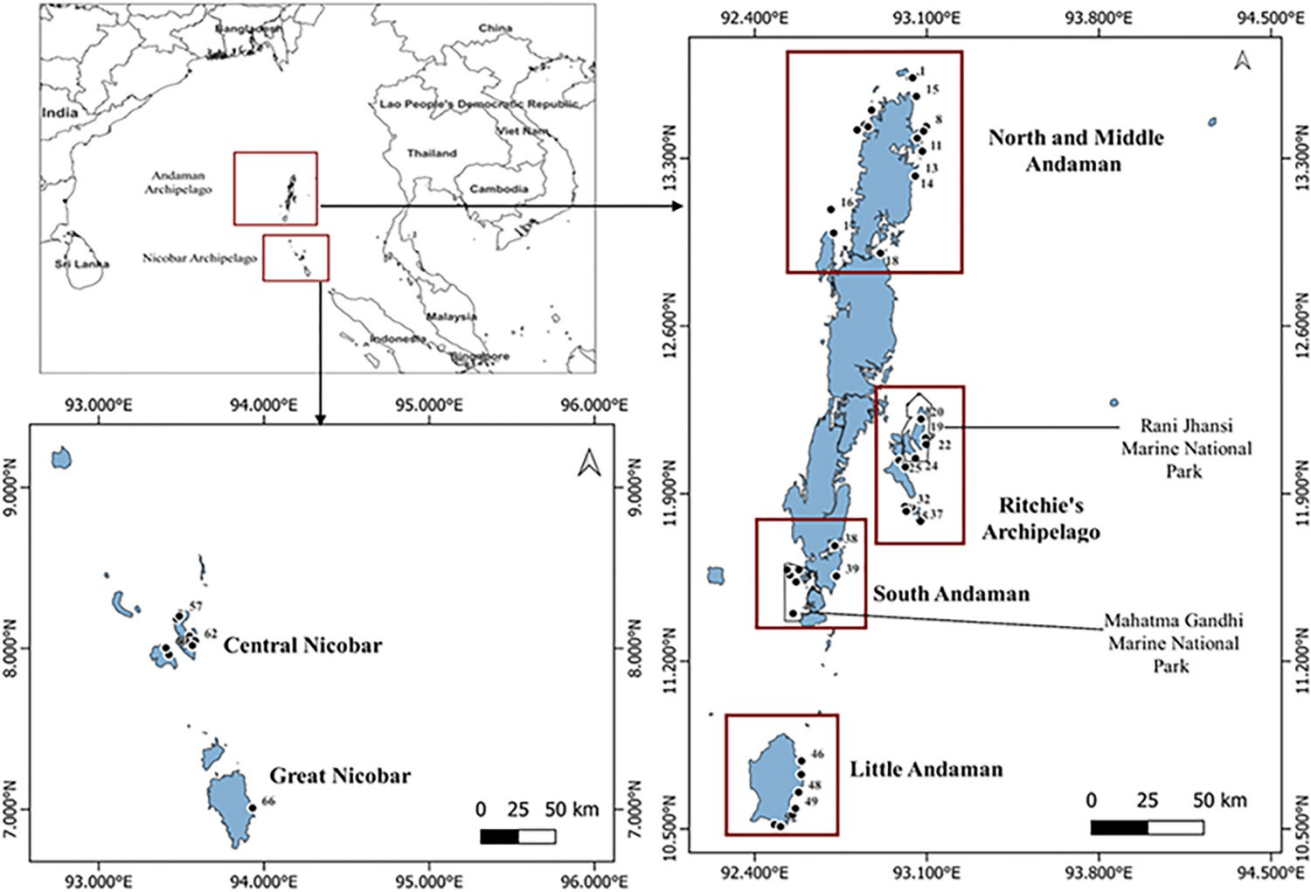
Study area map showing investigated seagrass locations in the Andaman and Nicobar Islands (see S1 Table for site names).

N&MA (13° 38’ 7.91" N to 12° 54’ 15.44" N latitude; sampling extent) forms the northernmost island mass of the Andaman archipelago. Sampling points in this group were spatially spread along the West, East, and North coast (Fig 1; sites 1–18). RA (12° 12’ 37.02" N to 11° 46’ 42.55" N; sampling extent) formed the second surveyed group (Fig 1; sites 19–37). RA is a cluster of 13 Islands on the eastern coast of the Andaman archipelago, including the Rani Jhansi Marine National Park (RJMNP). Sampling in SA (11° 41’ 15.32" N to 11° 23’ 03.86" N; sampling extent) was predominantly done in the second marine protected area of the Islands, the Mahatma Gandhi Marine National Park (MGMNP), with two locations outside the Marine National Park, on the south-eastern coast (Fig 1; sites 38–45). Further to the South, the fourth sampling cluster was LA (10° 48’ 15.07" N to 10° 29’ 39.32" N; sampling extent), a geographically isolated island at the transition between the Andaman and Nicobar archipelago. Sampling points here stretched from the east coast to South Bay (Fig 1; Sites 46–52). Lastly, the central Nicobar archipelago (8° 13’ 26.30" N to 6° 59’ 43.25" N; sampling extent), including four of the Nancowry group of Islands and Great Nicobar were treated as one latitudinal sampling cluster (Fig 1; Sites 53–66).

### Field sampling

We conducted five field surveys from January to April (2018 to 2022) to explore, locate and study seagrass meadows. Seagrass presence was confirmed at 66 meadows across five island groups (Fig 1), where we performed a detailed meadow characterization. Each sampling season aligned with calmer seas in the ANI, allowing thorough on-foot exploration in the intertidal habitats and SCUBA diving for subtidal surveys. We conducted seagrass exploratory surveys across the study sites’ depth (0.2 to 37 m) and latitudinal gradients; N&MA (n = 18 sites), RA (n = 19 sites), SA (n = 8 sites), LA (n = 7 sites), and NIC (n = 14 sites) (Fig 1).

We deployed 50 m long Line Intercept Transects [34] aligning perpendicular to the shore. A 0.5 X 0.5 m quadrat was treated as a sampling unit and placed on the 50 m transect line after every 5 m interval (11 sampling points/ transect). The quadrat was divided after every 10 cm (25 squares/ quadrat) to estimate seagrass species composition and percentage cover (total and species-specific). Species were confirmed by on-field photo-documentation and further referring to literature on seagrasses of ANI [35] and standard field guides [36]. We evaluated the percentage of seagrass cover by counting the total number of squares occupied by seagrasses (total and species-specific) in each quadrat. Each sampling unit’s (quadrat) data was averaged to give mean seagrass cover for each island group. We collected three samples per transect from a smaller 0.2 X 0.2 m quadrat to estimate the shoot densities for each species. Sampling locations were marked using Garmin Etrex 30.

### Data analysis

We tested the dissimilarity in seagrass richness and occurrence across the latitudinal gradient using the non-metric multi-dimensional scaling (NMDS) ordination method. To determine differences in seagrass community composition amongst investigated latitudinal gradients, we used permutational multivariate analysis of variance (PERMANOVA; 999 permutations) on the Jaccard’s dissimilarity matrix (species presence-absence data). Once we established significant differences in species composition and occurrence across the latitudinal gradient in PERMANOVA, we further used Multi-Response Permutation Procedure (MRPP) analysis. MRPP was performed to test for dissimilarities in species composition among the Island groups. MRPP was carried out with ten combinations ’between two Island groups’ to understand which two investigated Islands were more dissimilar in seagrass communities. All the analyses were performed in *R* (version 4.2.1) with extended *Vegan*, *MASS*, and *BiodiversityR* packages using *metaMDS*, *ordisymbol*, *adonis2*, and *mrpp* functions [37].

We determined the ecologically dominant species within the seagrass population for each Island group (latitudinal gradient) by calculating the Importance Value Index (IVI) based on the methods of Curtis & McIntosh [38]. For IVI estimation, we used the Relative frequency (RFi) and Relative density (RDi) of each species found within an island group using the following equation:

Importance Value Index (IVI) = RFi + RDi, where

Fi = occurrence of a species i in sampling plots/ Total number of sampling plots (quadrat)

RFi = frequency of species i/ Total frequency of all species (In each Island group) X 100

Di = density of species i / 0.04 m^2^

RDi = density of species i / Total density of all species (In each Island group) X 100

Calculating the relative dominance (basal area covered by an individual shoot) used for the IVI index is not feasible for herbaceous plants like seagrasses (as opposed to woody vegetation). Thus, we excluded relative dominance for this analysis. Instead, we considered RDi and RFi ecological measures representing species spread and dominance, as suggested by Rasingam & Parthasarathy [39]. Thus, the modified Importance Value Index here was a total of 200 for all seagrass species.

Lastly, we compared the present findings with earlier seagrass surveys with particular emphasis on pre-and post-tsunami status. We reviewed all published literature on seagrass spatial assessments in ANI from 1991 to 2018. For pre-tsunami studies, work done by Jagtap [40,41], and Das [33] was considered, where the collective data collection was done between 1990 to 1995. Similarly, for post-tsunami, assessments by Thangaradjou et al. [21,42], D’Souza et al. [43], Ragavan et al. [35] and Savurirajan et al. [44] were considered (collective study period was between 2007 to 2014), along with the present study (2018 to 2022). However, within these published studies, only sites with information on detailed localities (geographic coordinates/ site name/ island name) were used for comparison with our results. We removed seagrass meadows lacking the locality details, as mentioned earlier. A total of 12 sites collectively representing the investigated five latitudinal gradients were shortlisted for comparison, with a mandate of at least one pre-tsunami study as a baseline (Table 1). For these selected sites, pre- and post-tsunami variation in seagrass species composition was checked, to assess the temporal change in seagrass communities.

**Table 1:** A pre-and post-tsunami assessment of variation in species composition within each island group.

Sr. no.	Island group	Site name	Source [40,41] (1990)	Source [33] (1994–1995)	Source [21,42] (2010)	Source [43] (2007–2013)	Source [35] (2013–2014)	Source [44] (2013)	Present study (2018–2022)
1	N&MA	Kalipur	-	*Cr*^*j*^, *Th*^*a*^	*Cr*^*j*^, *Th*^*a*^	*Cr*^*j*^, *Th*^*a*^	-	-	*Cr*^*j*^, *Th*^*a*^, *Ea* ^b^*
2	N&MA	Interview		*Cr*^*j*^, *Th*^*a*^	-	-	-	-	*Hp* ^*g*^ *
3	N&MA	North Reef	-	*Cr*^*j*^, *Th*^*a*^, *Ea*^b^, *Ho*^c^	-	*Ho* ^c^	-	-	*Ho* ^c^
4	RA	Henry Lawrence	-	*Cr*^*j*^, *Ea*^b^, *Ho*^c^, *Hot*^*f*^, *Hp*^*g*^, *Hu*^*h*^, *Si*^*i*^, *Th*^*a*^	*Cr*^*j*^, *Ea*^b^, *Ho*^c^, *Hot*^*f*^, *Hp*^*g*^, *Hu*^*h*^, *Si*^*i*^, *Th*^*a*^	*Ea*^b^, *Ho*^c^, *Hp*^*g*^, *Hu*^*h*^, *Si*^*i*^, *Th*^*a*^	-	-	*Cr*^*j*^, *Ea*^b^, *Ho*^c^, *Hp*^*g*^, *Hu*^*h*^, *Si*^*i*^, *Th*^*a*^, *Hm*^e^*
5	RA	Swaraj Dweep (Havelock)	-	*Cr*^*j*^, *Ea*^b^, *Ho*^c^, *Hot*^*f*^, *Hp*^*g*^, *Hu*^*h*^, *Th*^*a*^	*Cr*^*j*^, *Ho*^c^, *Hot*^*f*^, *Hp*^*g*^, *Hu*^*h*^, *Th*^*a*^	*Ho*^c^, *Hp*^*g*^	*Cr*^*j*^, *Ea*^b^, *Ho*^c^, *Hp*^*g*^, *Hu*^*h*^, *Th*^*a*^, *Hm*^e^*	*Cr*^*j*^, *Ea*^b^, *Ho*^c^, *Hp*^*g*^, *Hu*^*h*^, *Th*^*a*^, *Hm*^e^	*Cr*^*j*^, *Ea*^b^, *Ho*^c^, *Hu*^*h*^, *Th*^*a*^, *Hm*^e^, *Cs*^k^*, *Si*^*i*^ *
6	SA	MGMNP	-	*Hot*^*f*^, *Hp*^*g*^, *Th*^*a*^	-	*Ho* ^c^ *	-	-	*Ho*^c^, *Hu*^h^*
7	LA	East and South coast	-	*Cr*^*j*^, *Ho*^c^, *Hp*^*g*^, *Hu*^*h*^, *Th*^*a*^	*Cr*^*j*^, *Th*^*a*^, *Cs*^k^*	*Cr*^*j*^, *Ho*^c^, *Hm*^e^*	*Cr*^*j*^, *Ho*^c^, *Hp*^*g*^, *Hu*^*h*^, *Th*^*a*^, *Hm*^e^, *Ea*^b^*	*Cr*^*j*^, *Ho*^c^, *Hu*^*h*^, *Th*^*a*^, *Ea*^b^	*Cr*^*j*^, *Ho*^c^, *Hp*^*g*^, *Hu*^*h*^, *Th*^*a*^, *Cs*^*k*^
8	NIC	Katchal	*Cr*^*j*^, *Ea*^b^, *Hu*^*h*^, *Hot*^*f*^, *Ho*^c^, *Si*^*i*^, *Th*^*a*^	*Cr*^*j*^, *Ea*^b^, *Hu*^*h*^, *Ho*^c^, *Si*^*i*^, *Th*^*a*^, *Cs*^*k*^ *, Hp*	-	*Hu*^*h*^, *Ho*^c^	*Cr*^*j*^, *Ea*^b^, *Hu*^*h*^, *Ho*^c^, *Si*^*i*^, *Hm*^e^*	-	*Hu*^*h*^, *Hp*^*g*^, *Ho*^c^, *Hm*^e^
9	NIC	Kamorta	-	*Cs*^*k*^, *Cr*^*j*^, *Ea*^b^, *Ho*^c^, *Hot*^*f*^, *Hp*^*g*^, *Hu*^*h*^, *Si*^*i*^, *Th*^*a*^	-	*Ea*^b^, *Ho*^c^, *Hp*^*g*^, *Hu*^*h*^, *Hm*^e^*	*Cr*^*j*^, *Ea*^b^, *Ho*^c^, *Hu*^*h*^, *Si*^*i*^, *Th*^*a*^, *Hm*^e^	-	*Cr*^*j*^, *Ea*^b^, *Ho*^c^, *Hp*^*g*^, *Hu*^*h*^, *Si*^*i*^, *Hm*^e^, *Hd*^d^*
10	NIC	Nancowry	*Cr*^*j*^, *Ea*^b^, *Hu*^*h*^, *Hot*^*f*^, *Ho*^c^, *Si*^*i*^, *Th*^*a*^	*Cr*^*j*^, *Ea*^b^, *Hu*^*h*^, *Ho*^c^, *Si*^*i*^, *Th*^*a*^, *Cs*^k^*, *Hp*^g^*	-	*Cr*^*j*^, *Ea*^b^, *Hu*^*h*^, *Ho*^c^, *Si*^*i*^, *Th*^*a*^, *Cs*^*k*^, *Hp*^*g*^	*Cr*^*j*^, *Ea*^b^, *Hu*^*h*^, *Ho*^c^, *Si*^*i*^, *Th*^*a*^, *Cs*^*k*^, *Hm*^e^*	-	*Cr*^*j*^, *Ea*^b^, *Hu*^*h*^, *Ho*^c^, *Si*^*i*^, *Th*^*a*^, *Cs*^*k*^, *Hp*^*g*^, *Hm*^e^
11	NIC	Trinket	-	*Cr*^*j*^, *Cs*^*k*^, *Ea*^*b*^, *Ho*^c^, *Hot*^*f*^, *Hu*^*h*^, *Hp*^*g*^, *Si*^*i*^, *Th*^*a*^	-	*Hm* ^e^ *	-	-	*Cr*^*j*^, *Ea*^b^, *Ho*^c^, *Hu*^*h*^, *Hp*^*g*^, *Si*^*i*^, *Th*^*a*^
12	NIC	Great Nicobar	*Cr*^*j*^, *Hu*^*h*^, *Th*^*a*^	*Cr*^*j*^, *Th*^*a*^, *Ea*^b^*, *Si*^i^*	*Cr*^*j*^, *Hu*^*h*^, *Th*^*a*^, *Ea*^*b*^, *Si*^*i*^ *Cs*^k^*, *Ho*^c^*, *Hot*^f^*, *Hp*^g^*	*Hp* ^*g*^	*Cr*^*j*^, *Hu*^*h*^, *Th*^*a*^, *Ho*^c^, *Hm*^e^*	-	*Hu*^*h*^, *Hp*^*g*^, *Ho*^c^, *Hm*^e^

*New species distribution record for the region; ^a^*Thalassia hemprichii*, ^b^*Enhalus acoroides*, ^c^*Halophila ovalis*, ^d^*Halophila decipiens*, ^e^*Halophila minor*, ^f^*Halophila ovata*, ^g^*Halodule pinifolia*, ^h^*Halodule uninervis*, ^i^*Syringodium isoetifolium*, ^j^*Cymodocea rotundata*, ^k^*Cymodocea serrulata;* N&MA, The North and Middle Andaman; RA, The Ritchie’s archipelago; SA, The South Andaman; LA, The Little Andaman; NIC, The Nicobar Islands

## Results

### Variation in the seagrass species richness across a latitudinal gradient

**Fig 2:**
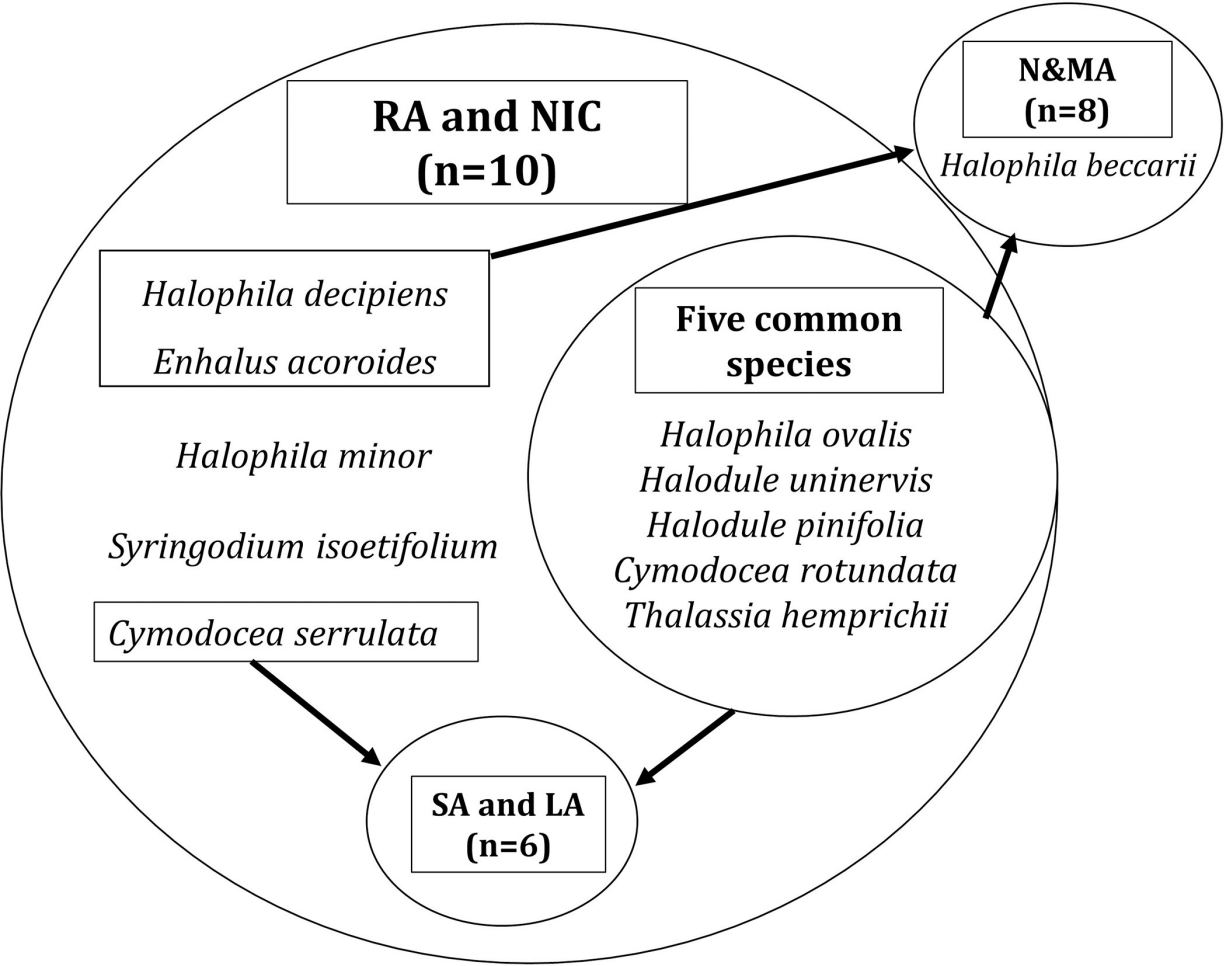
Schematic representation of seagrass species communities across investigated island groups.

A total of 11 species of seagrasses belonging to the families Cymodoceaceae (n = 5) and Hydrocharitaceae (n = 6) were observed during the study period (Fig 2). Five of these species, *Halophila ovalis*, *Halodule uninervis*, *Halodule pinifolia*, *Cymodocea rotundata*, and *Thalassia hemprichii* commonly occurred across all the latitudinal gradients (Fig 2). RA and NIC formed the largest group of species richness and a similar species composition (Fig 2). A subset of ’LA and SA’ was further formed within the larger ’RA and NIC’ set, with six common species across four island groups. (Fig 2). Seven species were shared between N&MA and ’RA, and NIC’ except for *Halophila beccarii*, which was exclusive to N&MA (Fig 2).

**Fig 3:**
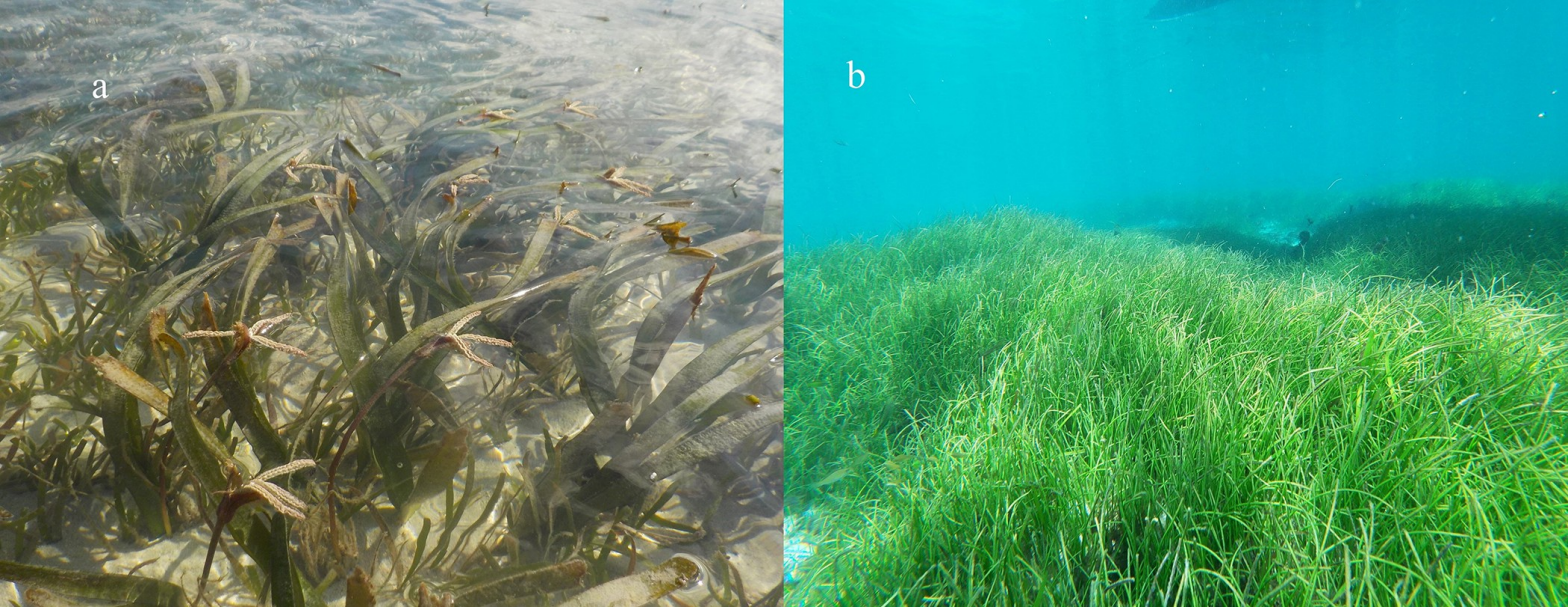
a) *Enhalus acoroides* dominated mixed-species intertidal meadow, Swaraj Dweep (RA) (picture credit- Swapnali Gole), b) *Syringodium isoetifolium* dominated mixed-species shallow subtidal meadow, Nancowry (NIC) (picture credit- J.A. Johnson).

**Fig 4:**
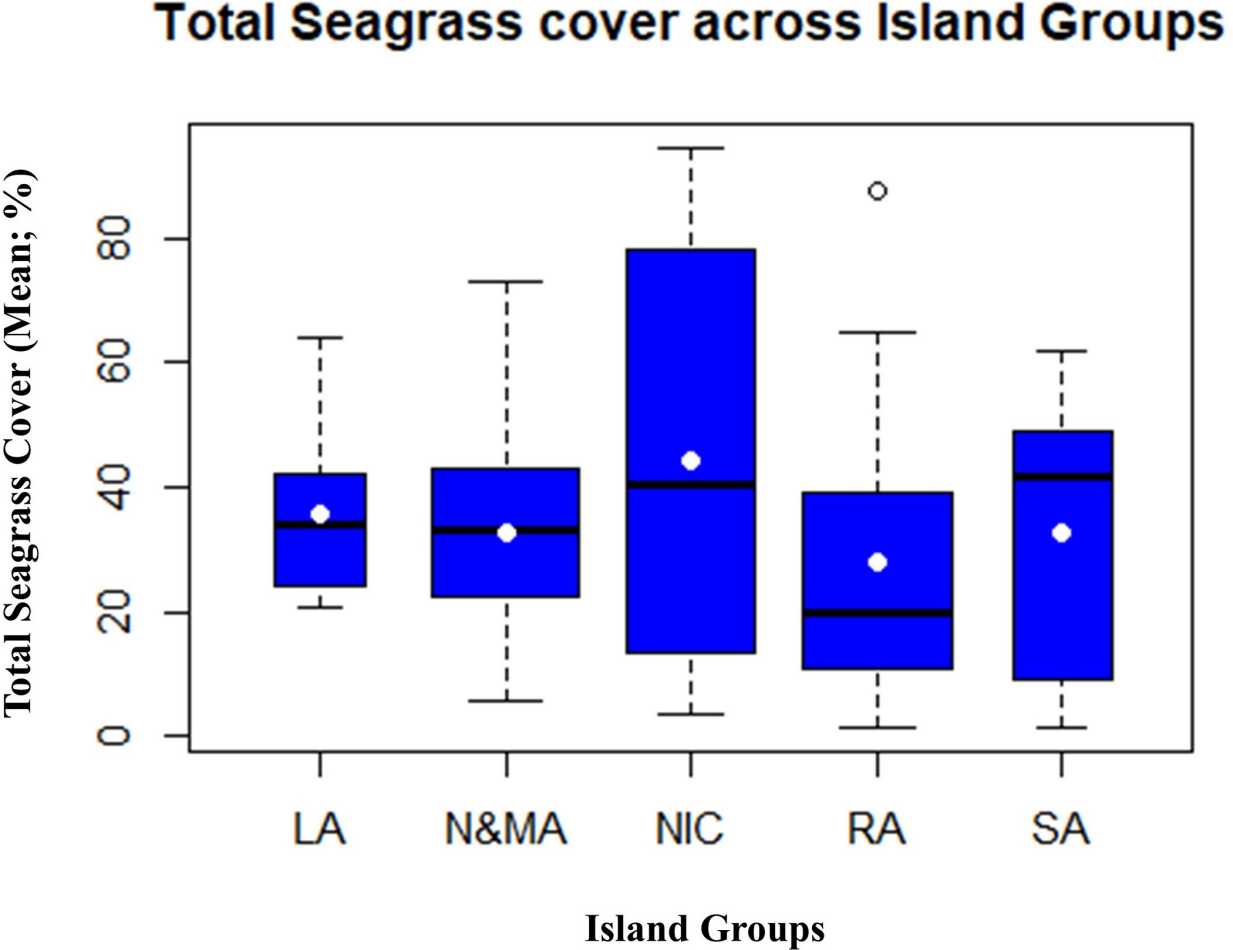
Boxplots showing the dispersion of seagrass cover across the investigated latitudinal gradients. The box’s black line and white circle denote the median and mean values, respectively.

Seagrass meadows in the N&MA, SA, and NIC occurred in the intertidal and shallow subtidal habitats (< 8 m; Fig 3). Seagrass communities in LA were restricted to intertidal regions. RA had a more dynamic depth gradient in species distribution, spreading across intertidal, shallow subtidal, and deep waters (up to 21m). A noteworthy record in the present study was *Halophila decipiens* from 21 m observed at site Busy Buro, Shaheed Dweep in RA (Fig 1; Site 28). Seagrass cover varied in dispersion amongst all the Island groups. NIC group had the most widely spread seagrass cover, followed by SA (Fig 4). Further, LA and N&MA showed similar trends, where both groups had the least dispersion of seagrass cover (Fig 4). Seagrass communities of RA and NIC showed significant coverage variation, despite their species composition similarity. NIC had the highest total seagrass cover (mean), while the lowest cover was recorded from RA (Fig 4).

**Fig 5:**
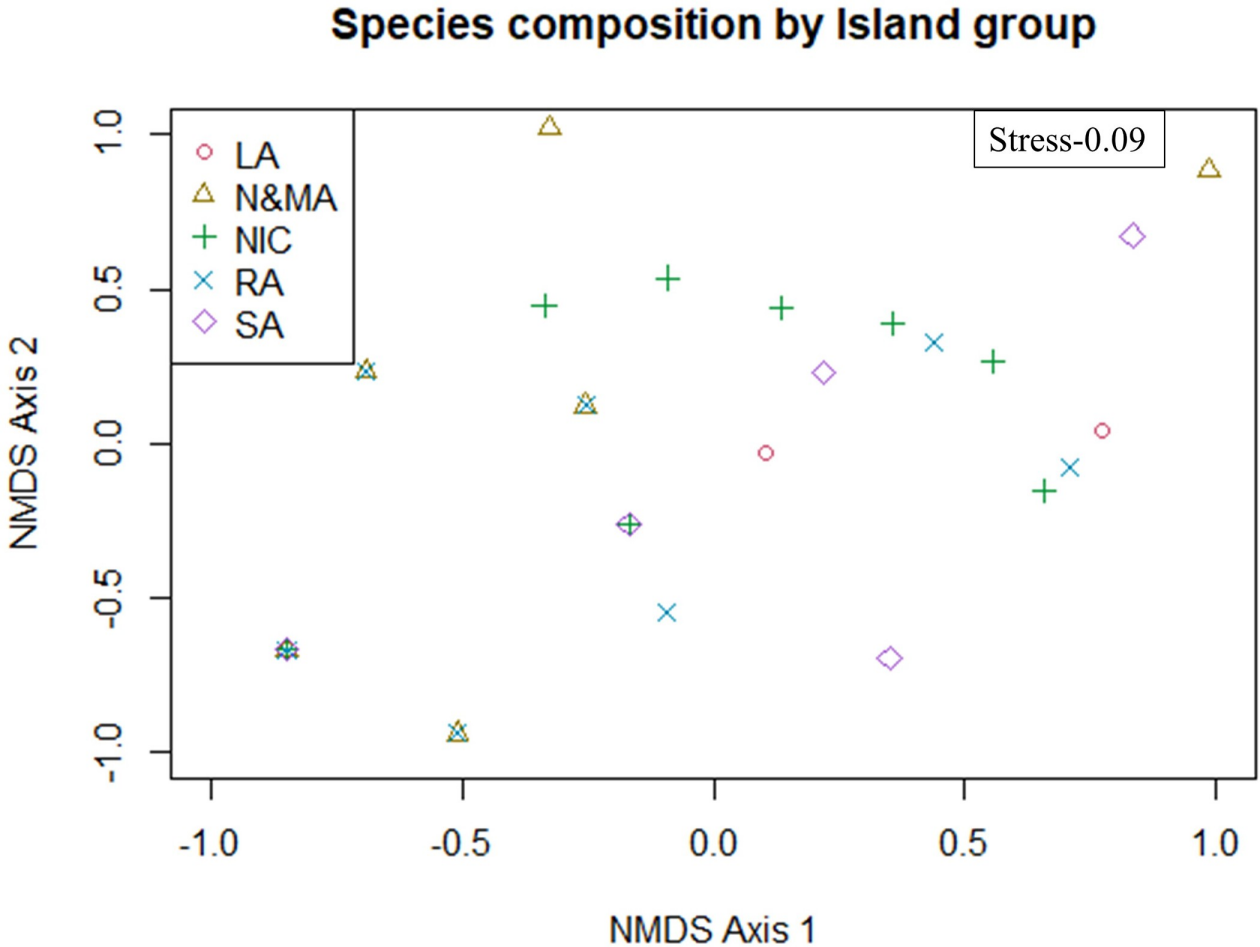
NMDS findings on species diversity across investigated five island groups. Sites placed closer have similarities in species richness.

**Fig 6:**
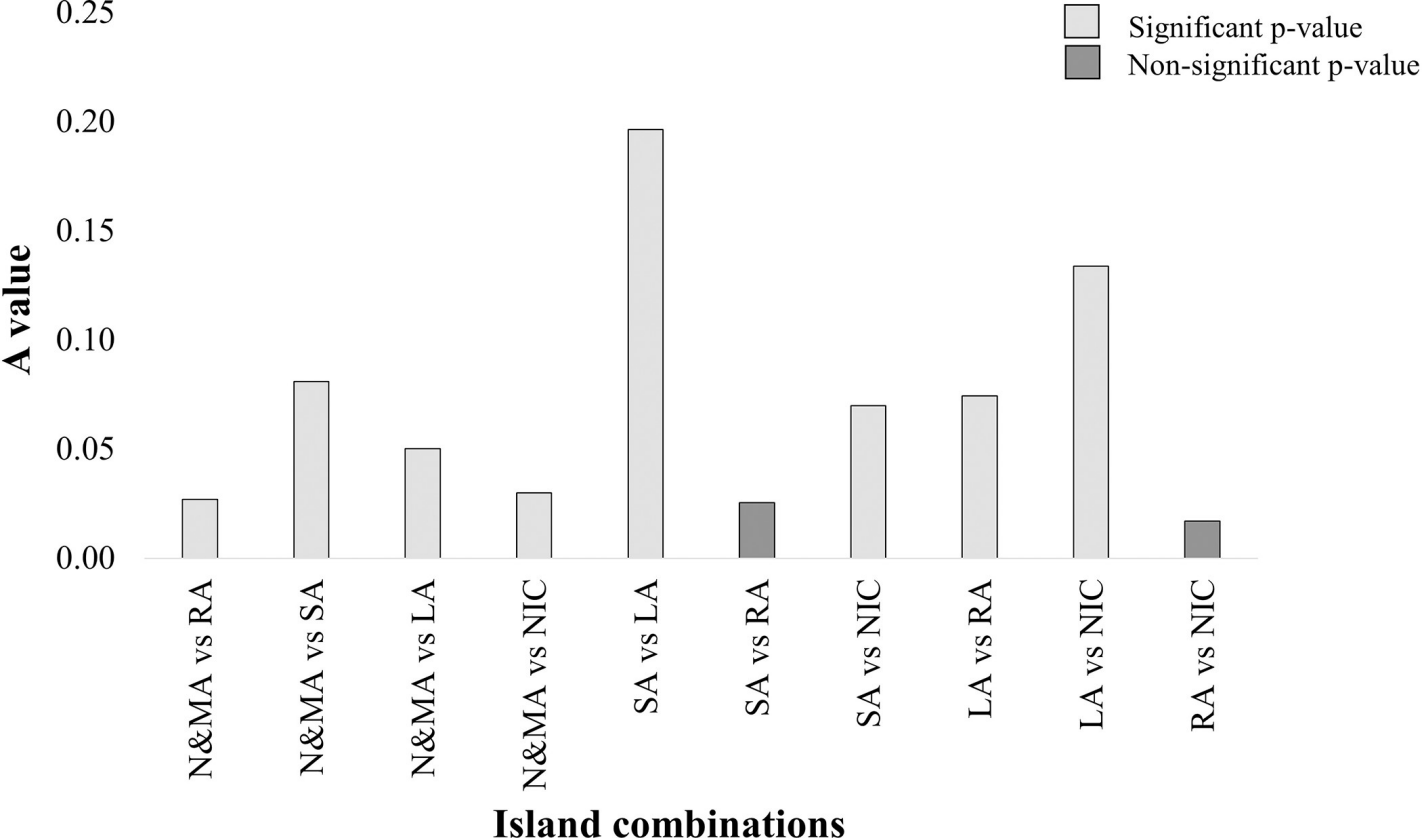
MRPP results show dissimilarities in seagrass species diversity across different island types.

We observed a significant variation in seagrass species richness across the latitudinal gradient of the five investigated Island groups (NMDS and PERMANOVA: *F*- 3.24, *p*-value- 0.001, R^2^-0.18; Fig 5). Island groups with similar species richness, ’SA and LA’ revealed the least variation in community composition (Fig 6 and Table 2). On the contrary, seagrass populations in RA and NIC with the same species richness exhibited the highest dissimilarity in community composition (Fig 6 and Table 2). Communities in ’N&MA with RA and NIC’ and ’SA with RA’ shared distantly related seagrass species with significant dissimilarities (Fig 6 and Table 2).

**Table 2:** Results of Multi-Response Permutation Procedure (MRPP) analysis performed on species composition across island categories.

Site combination	A	P	observed delta	expected delta
**Whole data**	0.09	0.001	0.6885	0.7603
**N&MA vs RA**	0.03	0.026	0.7298	0.75
**N&MA vs SA**	0.08	0.005	0.623	0.6778
**N&MA vs LA**	0.05	0.047	0.6438	0.6778
**N&MA vs NIC**	0.03	0.03	0.6943	0.7157
**SA vs LA**	0.20	0.002	0.5455	0.6787
**SA vs RA**	0.03	0.107	0.7141	0.7327
**SA vs NIC**	0.07	0.013	0.6589	0.7084
**LA vs RA**	0.07	0.007	0.7303	0.789
**LA vs NIC**	0.13	0.001	0.6763	0.7807
**RA vs NIC**	0.02	0.091	0.7667	0.78

N&MA, The North and Middle Andaman; RA, The Ritchie’s archipelago; SA, The South Andaman; LA, The Little Andaman; NIC, The Nicobar Islands.

**Fig 7:**
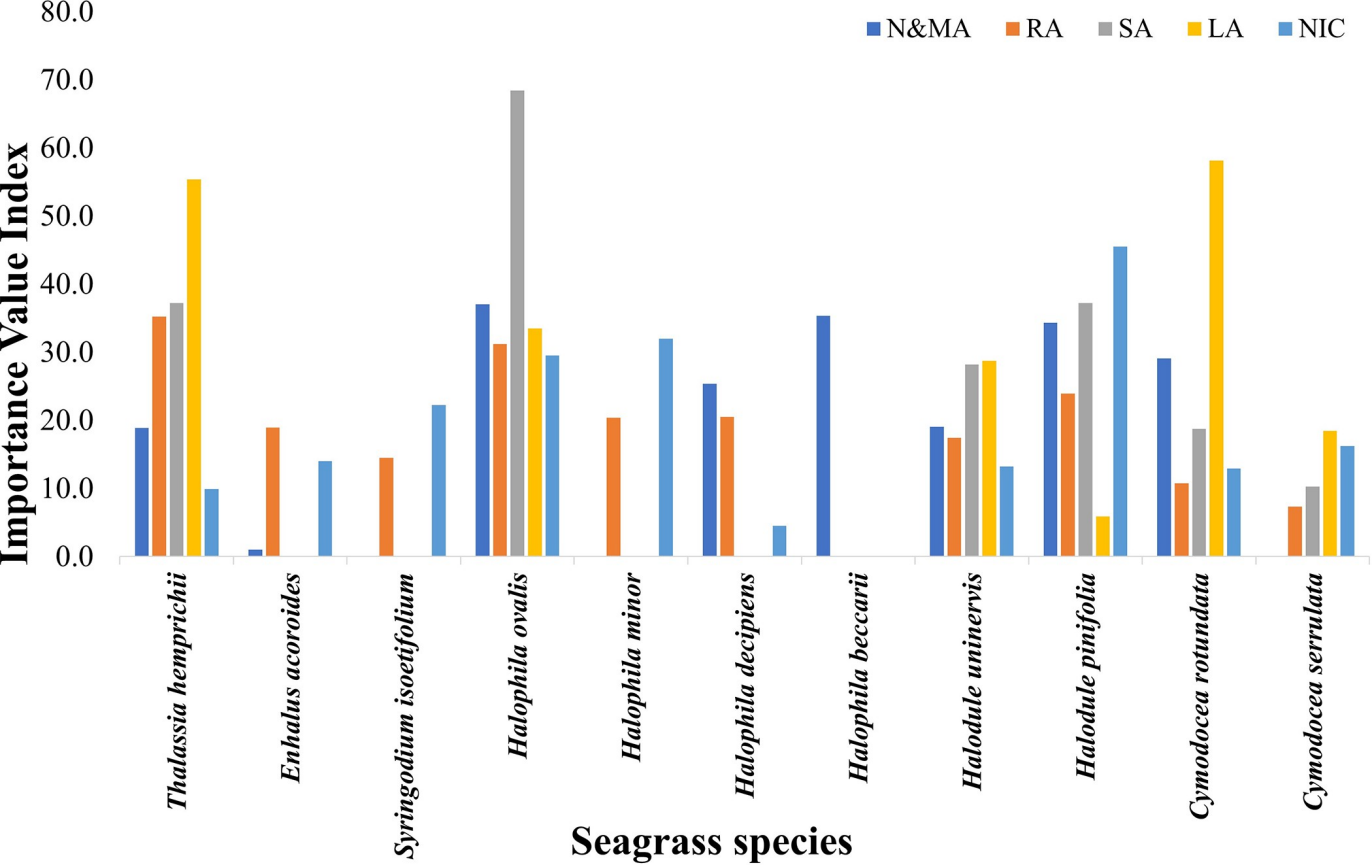
Ecologically dominant seagrass species in terms of the Importance Value Index (IVI) within each investigated island group.

We identified six dominant seagrass species among all the Island groups (Fig 7). *H*. *ovalis*, *H*. *beccarii*, and *Halodule pinifolia* were dominant species in N&MA, with 37%, 35.3%, and 34.3% contributions, respectively (Fig 7). *T*. *hemprichii* and *H*. *ovalis* largely occupied the seagrass populations in the RA and SA. In addition to these two species, *Halodule pinifolia* was dominant in SA (Fig 7). We abundantly observed *C*. *rotundata* and *T*. *hemprichii* in the intertidal meadows LA while *Halodule pinifolia*, and *Halophila minor* in NIC (Fig 7).

### Variation in the seagrass species composition within each island group: Special emphasis on pre-and post-tsunami perspective

Seagrass communities in RA and LA showed a stable pre- and post-tsunami species composition. Meanwhile, it varied for SA, N&MA, and NIC (Table 1). We observed similar seagrass species in the intertidal meadow at Kalipur (Fig 1; Site 14), with the addition of *Enhalus acoroides* as a new record (Table 1). However, mono-specific *Halodule pinifolia* and *H*. *ovalis* meadows replaced the pre-tsunami species composition at Interview Island and North Reef, N&MA (Fig 1; Sites 16 and 17) (Table 1). Temporal seagrass composition was almost similar in the LA and RA, with new distribution records of *Cymodocea serrulata* and *Syringodium isoetifolium* from Swaraj Dweep (Table 1). We though, did not observe *H*. *minor* and *E*. *acoroides* from LA in the present study. Seagrass meadows in the Mahatma Gandhi Marine National Park, South Andaman, exhibited a changed species composition (Fig 1 and Table 1). The present seagrass community of MGMNP was characterized by *H*. *ovalis* and *Halodule uninervis* as opposed to pre-tsunami records of *Halophila ovata*, *Halodule pinifolia*, and *T*. *hemprichii* (Table 1).

Pre- and post-disturbance seagrass status in the Nancowry and Trinket islands remained largely unchanged. At the same time, composition varied in Kamorta and Katchal islands (Fig 1 and Table 1). *C*. *serrulata* and *H*. *ovata* from Kamorta were not observed in any study after the tsunami, counting our work. In addition, we report a new observation of *H*. *decipiens* from Kamorta (Table 1). In Katchal, we observed only two out of eight seagrass species reported before the tsunami (Table 1). Later, *H*. *minor*, a new addition from the same region, was also observed in the present study (Table 1). All the species observed from Great Nicobar are synchronous with recent seagrass assessments except for *T*. *hemprichii* and *C*. *rotundata* (Table 1). Lastly, one-time records of *C*. *serrulata* and *H*. *ovata* from the Great Nicobar Island were unreported in other seagrass assessments (Table 1).

## Discussion

Globally, studies on latitudinal variation in species diversity have focused on larger geographical spaces (across hemispheres or ocean basins) [2,45]. Yet the local patterns of variation that drive community composition and traits, though crucial [10], remain underexplored. Our study attempted to document seagrass community variation locally and suggests a substantial variability across the investigated latitudinal gradients. Although the species richness was comparable, the ecologically dominant species and seagrass coverage differed amongst Island groups. We suggest that, latitudinal variation may have led to distinct geographical clusters of seagrass communities in the Islands, and could be driven by the local environment. Along the north to south latitudinal gradient, seagrasses of three island groups showed affinities in different degrees; SA with LA, and LA with NIC. This, possibly is a result of LA being geographically transitioned between SA and NIC, acquiring environmental traits from both. We further suggest that, distinct island clusters such as RA and NIC, owing to their remoteness, indicate higher separation of seagrass communities from the rest of the groups. Seagrass assemblages in RA showed no coherence with almost all island groups, indicative of a micro-habitat unique to this island group. Further, seagrasses in N&MA (the northernmost latitude) and SA too, exhibit greater separation from NIC (the southernmost latitude). The understanding of this segregation and similarities between seagrass communities in ANI, is essential to know how seagrasses would respond to changing local environment, especially when small-scale stress and disturbances are known to impact the plant performance and growth [46].

Variation in species occurrence, dominance, along with seagrass coverage observed could be attributed to the potential co-variables associated with latitude as a surrogate, such as a habitat heterogeneity, substratum availability, water depth, topography, nutrient dynamics, and wave exposure [44,47,48]. For example, despite having the highest species richness, NIC and RA exhibited varying seagrass cover. A recent study in the Islands, suggests the critical depth limit of seagrass growth to be ~ 2 to 5 m, after which seagrass coverage, biomass and shoot densities were reported to decline [49]. Water depth impacts light availability, photosynthesis, growth and therefore influences seagrass cover [11]. Furthermore, each seagrass species has different thresholds and saturating irradiance for photosynthesis, which directly affects seagrass distribution [47,50]. Therefore, water depth could be a possible determinant of low seagrass coverage in RA, since > 70% of seagrass meadows of this island group were shallow to deep subtidal in distribution (n = 14). At the same time, meadows in NIC occurred largely in shallow waters, and could have conducive co-variates associated with water depth, supporting high coverage. Moreover, our observations on seagrasses from 21 m further highlight the potential of deep-water seagrasses in the ANI, and the need for further exploration, as seagrass studies in the islands have majorly focused on the shallow waters [21,35,42,44].

Pre- and post-tsunami comparison revealed three trends: a) local extinction of few seagrass species, b) re-colonization of disturbed areas by new species, and c) re-colonization of disturbed areas by historically distributed species. In addition, the disturbed areas were recolonized mainly by the early successional species of the genera, *Halophila* spp. and *Halodule* spp. (Table 2) [51,52]. *H*. *ovata*, reported primarily in pre-tsunami studies [33,41], was never observed in any post-tsunami study, except by Thangaradjou et al. [21]. Post-tsunami ground surveys [21] reported the absence of seagrasses from the Interview and North Reef Islands in N&MA, where four species were reported pre-disturbance [33]. A total reported seagrass denudation of respective ~ 124 ha and 23 ha from these islands [53] was mainly because of the transition of seagrass habitats into dead coral reefs post the coastal uplift following the earthquake of 2004 [21]. After nearly three decades, contrary to Das’s [33] observations of *C*. *rotundata* and *T*. *hemprichii*, we report *Halodule* spp. colonizing the shallow waters of post-tsunami seagrass-denuded regions of Interview Island and North Reef islands [53]. These observations suggest the revival of a conducive environment for seagrass colonization in the area. Similarly, we confirm the shift in the species composition observed in the North Reef Island, N&MA, from four species reported earlier [33] to mono-specific *H*. *ovalis* beds as observed by D’Souza et al. [43].

Prior seagrass studies have highlighted RA, as a diverse seagrass region, with high species richness [21,33,35,43,44,53]. Our work confirms the same, with additions of *H*. *minor* from Henry Lawrence, *C*. *serrulata*, and *S*. *isoetifolium* from Swaraj Dweep to the region’s checklist. In the MGMNP (SA), the late-successional species *T*. *hemprichii* [54] reported by Das [33] was replaced by *Halodule uninervis* observed in the present work, in addition to *H*. *ovalis* reported after the tsunami [43]. In LA, low seagrass richness (three species) was reported till a decade after the tsunami in 2004, with new records of *C*. *serrulata* and *H*. *minor* [21,43] unreported earlier [33]. Our observations report six species from the region. They agree with recent assessments [35,44], which collectively indicate the re-colonization of historic species in the region.

Further, we observed a noteworthy change in the seagrass status of the Nancowry group of Islands, NIC. It is apparent from the literature that seagrass communities in the Nicobar Islands were rich pre-tsunami [33,41]. However, the Sumatra-Andaman Earthquake and subsequent tsunami significantly impacted the seagrass habitats in the Nicobar Islands [53] along with mangroves and coral reefs [28,55]. Nevertheless, the pre-and post-tsunami assessments, complemented by our findings, suggest a seagrass population recovery in the NIC. In Katchal Island, seagrass community composition has drastically varied in a pre-and post- tsunami scenario. We observed four early successional species of the genera *Halophila* spp. and *Halodule* spp. [25] as opposed to eight early and late successional species reported by Das [33], dominating the seagrass-bared regions of Katchal [53]. Trinket, once a seagrass-rich region [33], remained one of the worst-hit islands by tsunami [23]. A recent seagrass survey by D’Souza et al. [43] observed only *H*. *minor*, an early-successional species from the region. On the contrary, after ~ two decades of post-tsunami, we observed seven out of nine species reported by Das [33]. Similar observations from Nancowry and Kamorta concur with pre-tsunami baseline studies [33,41], suggesting the re-colonization of disturbed areas by early successional and historically distributed species in NIC.

Global studies have highlighted similar seagrass re-colonization trends following a disturbance. Early colonizers, like *Halodule* spp. and *Halophila* spp., are fast-growing plants with higher metabolic rates and greater dispersal abilities [56,57]. As a result, these species can outcompete late-successional species in case of heavy nutrient loading in the environment [57,58], which was evident in the post-tsunami scenario in the ANI. Further, the early and late successional species exhibit different growth strategies following a major disturbance [25]. Early colonizers are more susceptible to disturbance and removed from a meadow. At the same time, the late-successional species have a more resilient architecture (robust below-ground structures) to withstand stress [59,60]. However, post-disturbance fast re-colonization abilities of early successional species allow them to occupy bare sediments and niches dominated by the competitive late-successional species [16,52,60]. This probably explains the change in the ecological dominance of seagrass species in the Nicobar archipelago from *T*. *hemprichii* and *C*. *rotundata*, as observed by Das [33], to *Halodule pinifolia* and *H*. *minor* in our study. Lastly, the new species distribution records could also result from a rise in seagrass research and exploration in ANI after the tsunami and local spatio-temporal variations in the drivers that influence species distribution.

## Conclusion

Although several seagrass surveys in the ANI have focused on spatial diversity assessments, to our knowledge, this is the first attempt to study the seagrass community variation using latitude as a surrogate variable. Although, with significant dissimilarities, our findings suggest that latitude is not causation of the observed dissimilarity. However, it is a potential surrogate for ecological and environmental gradients (water depth, topography, habitat heterogeneity, nutrient dynamics) between the Island groups, which warrants further investigation. With the highest species richness, seagrass cover, and re-colonization by historic species at a local scale, we suggest that seagrass populations like mangroves [61] are reviving from the disturbance caused by the tsunami, especially in the Nicobar archipelago. In addition, new distribution records of species, including from deep waters, highlight the importance and need for continuous seagrass exploratory surveys to understand the species’ compositional dynamics after a significant disturbance event like the 2004 tsunami.

## Supporting information

S1 Table List of 66 seagrass meadows investigated across five latitudinal gradients in the Andaman and Nicobar archipelago. (DOCX)
