# Supplementary material for: Latitudinal variation in seagrass communities with special emphasis on post-tsunami status in the Andaman and Nicobar archipelago, India
Source: PLoS One. 2024 Mar 20;19(3):e0300654. doi: 10.1371/journal.pone.0300654 (PMC10954190; doi:10.1371/journal.pone.0300654)
Supplement: S1 Table — (DOCX) [file pone.0300654.s001.docx]

**S1 appendix: List of 66 seagrass meadows investigated across five latitudinal gradients in the Andaman and Nicobar archipelago**

| **Island Category** | **Site code** | **Site name** |
| --- | --- | --- |
| North & Middle Andaman | Site 1 | Landfall (South-East) |
| North & Middle Andaman | Site 2 | Landfall-East (Channel) |
| North & Middle Andaman | Site 3 | Reef (East) |
| North & Middle Andaman | Site 4 | Reef (South) |
| North & Middle Andaman | Site 5 | Paget (North-East) |
| North & Middle Andaman | Site 6 | Point (North) |
| North & Middle Andaman | Site 7 | Radhanagar Channel (Opening) |
| North & Middle Andaman | Site 8 | Excelsior (West) |
| North & Middle Andaman | Site 9 | Delgarno (West) |
| North & Middle Andaman | Site 10 | Temple (West) |
| North & Middle Andaman | Site 11 | Smith (North-East) |
| North & Middle Andaman | Site 12 | Smith (East) |
| North & Middle Andaman | Site 13 | Kohassa (Shibpur) |
| North & Middle Andaman | Site 14 | Kalipur |
| North & Middle Andaman | Site 15 | Craggy |
| North & Middle Andaman | Site 16 | North Reef (East) |
| North & Middle Andaman | Site 17 | Jhau Nala (Interview, North) |
| North & Middle Andaman | Site 18 | Pokkadera (Mayabunder) |
| Ritchie's archipelago | Site 19 | Imli Dera (Henry Lawrence- Shallow, RJMNP, North) |
| Ritchie's archipelago | Site 20 | Imli Dera (Henry Lawrence- deeper, RJMNP, North) |
| Ritchie's archipelago | Site 21 | Jua Tekdi (Henry Lawrence, RJMNP, East) |
| Ritchie's archipelago | Site 22 | Inglis (RJMNP, South-West) |
| Ritchie's archipelago | Site 23 | Chand Balu (Henry Lawrence, RJMNP, South-East) |
| Ritchie's archipelago | Site 24 | John Lawrence (RJMNP, South) |
| Ritchie's archipelago | Site 25 | Nemo Reef (Swaraj Dweep, North) |
| Ritchie's archipelago | Site 26 | Dolphin (Swaraj Dweep, North-East) |
| Ritchie's archipelago | Site 27 | Vijay Nagar (Swaraj Dweep, North-East) |
| Ritchie's archipelago | Site 28 | Busy Buro (Shaheed Dweep, North) |
| Ritchie's archipelago | Site 29 | Bharatpur (Shaheed Dweep-deep subtidal, North) |
| Ritchie's archipelago | Site 30 | Bharatpur (Shaheed Dweep-shallow subtidal, North) |
| Ritchie's archipelago | Site 31 | Aquarium (Shaheed Dweep-deep subtidal, North) |
| Ritchie's archipelago | Site 32 | Aquarium (Shaheed Dweep-shallow subtidal, North) |
| Ritchie's archipelago | Site 33 | Laxmanpur (Shaheed Dweep, North-West) |
| Ritchie's archipelago | Site 34 | Nursery (Shaheed Dweep, North-West) |
| Ritchie's archipelago | Site 35 | Natural Bridge (Shaheed Dweep, South-West) |
| Ritchie's archipelago | Site 36 | Macchi Gadda (Shaheed Dweep, South-West) |
| Ritchie's archipelago | Site 37 | Chota Neil (Sir Hugh Ross, West) |
| South Andaman | Site 38 | Haddo (Port Blair, East) |
| South Andaman | Site 39 | Burmanallah (Port Blair, East) |
| South Andaman | Site 40 | Chester (MGMNP, South-East) |
| South Andaman | Site 41 | Tarmugli (MGMNP, West) |
| South Andaman | Site 42 | Tarmugli (MGMNP, East) |
| South Andaman | Site 43 | Tarmugli (MGMNP, South) |
| South Andaman | Site 44 | Boat (MGMNP, East) |
| South Andaman | Site 45 | Twins (MGMNP, North) |
| Little Andaman | Site 46 | Patthar Nali (Little Andaman, East) |
| Little Andaman | Site 47 | Dungi Ghat (Little Andaman, East) |
| Little Andaman | Site 48 | Kalapatthar (Little Andaman, East) |
| Little Andaman | Site 49 | Hutbay Breakwater Jetty (Right-Little Andaman, South-East) |
| Little Andaman | Site 50 | Harminder Bay (Little Andaman, South-East) |
| Little Andaman | Site 51 | Hutbay-Lighthouse (Little Andaman, South) |
| Little Andaman | Site 52 | Ship Wreck (Little Andaman, South) |
| Nicobar | Site 53 | Kardip (Kamorta, South-East) |
| Nicobar | Site 54 | Bada Inak (Kamorta, South-East) |
| Nicobar | Site 55 | Rani Tikri (Kamorta, South) |
| Nicobar | Site 56 | Pilpilow (Rai-Kin-Fimo; Kamorta, North) |
| Nicobar | Site 57 | Pilpilow (Kaloweek; Kamorta, North) |
| Nicobar | Site 58 | Pilpilow (Moh-Ryak; Kamorta, North) |
| Nicobar | Site 59 | Hypo (Katchal, East) |
| Nicobar | Site 60 | Kapanga (Katchal, North-East) |
| Nicobar | Site 61 | Lighthouse (Katchal, North-East) |
| Nicobar | Site 62 | Safed Balu (Trinket, North) |
| Nicobar | Site 63 | Trinket (West coast, South-West) |
| Nicobar | Site 64 | Altaiyak (Nancowry, North) |
| Nicobar | Site 65 | Hitui (Nancowry, North) |
| Nicobar | Site 66 | Campbell Bay (Great Nicobar, East) |
